# Bushen Huatan Formula Combined with Conventional Therapy for Polycystic Ovary Syndrome: A Systematic Review and Meta-analysis

**DOI:** 10.2174/0118715303421221251201084638

**Published:** 2026-03-30

**Authors:** Yaru Wang, Qingrui Zhang, Menghan Jin, Shuai Zhang

**Affiliations:** 1 College of Traditional Chinese Medicine, Shandong University of Traditional Chinese Medicine, Jinan, China

**Keywords:** Polycystic ovary syndrome, Bushen Huatan formula, conventional therapy, infertility, traditional Chinese medicine, testosterone

## Abstract

**Introduction:**

Clinical research suggests that the Bushen Huatan Formula (BHF) may effectively improve polycystic ovary syndrome (PCOS)-related symptoms and infertility. However, no systematic summary of evidence currently exists. This systematic review was designed to assess the therapeutic efficacy and safety profile of BHF when used as a complementary treatment modality for PCOS.

**Methods:**

A systematic search was conducted across multiple databases, including the Cochrane Library, Embase, PubMed, Web of Science, China National Knowledge Infrastructure (CNKI), Wanfang Database, and the China Science and Technology Journal Database (VIP), up to 20 June 2024. Eligible randomized controlled trials (RCTs) included female patients diagnosed with PCOS and compared BHF treatment with conventional biomedical therapies. Results were pooled using random- or fixed-effects models, with effect sizes reported as odds ratios (ORs) or standardized mean differences (SMDs) and their 95% confidence intervals (CIs).

**Results:**

A meta-analysis of 11 RCTs involving 1,006 women with PCOS showed that combining BHF with conventional treatments significantly increased pregnancy rates (OR = 2.58, 95% CI = 1.97 to 3.38, *P* < 0.01) and ovulation rates (OR = 3.55, 95% CI = 2.29 to 5.48, *P* < 0.01). It also improved endometrial thickness (SMD = 0.85, 95% CI = 0.39 to 1.31, *P* < 0.01).

**Discussion:**

BHF had no significant effect on serum levels of total testosterone (T), luteinizing hormone (LH), follicle-stimulating hormone (FSH), or estradiol (E2). No serious adverse reactions were reported.

**Conclusion:**

The combination of BHF and conventional medications significantly improved pregnancy and ovulation rates, as well as endometrial thickness, in PCOS patients compared to conventional treatment alone. This suggests that BHF could serve as a complementary therapy for infertility in PCOS patients. However, these findings should be interpreted with caution due to significant heterogeneity and methodological limitations in the included studies.

## 1 INTRODUCTION

Polycystic Ovary Syndrome (PCOS) is a prevalent endocrine disorder among women of reproductive age, characterized by irregular menstruation, hyperandrogenism, and polycystic ovarian morphology [1]. Global prevalence varies widely, ranging from 2% to 21%, depending on diagnostic criteria [2]. Key contributors to PCOS development include insulin resistance (IR), hormonal dysregulation, and chronic low-grade inflammation, which are also associated with long-term complications such as type 2 diabetes mellitus (T2D), hypertension, non-alcoholic fatty liver disease, metabolic syndrome, and cardiovascular disease [3]. A meta-analysis revealed that women with PCOS are more likely to experience cardiometabolic risks, including T2D and hypertension, compared to non-PCOS women [4]. Early diagnosis can improve symptoms and reduce long-term risks. Women with PCOS frequently seek treatment for infertility, acne, and hirsutism. Approximately 25% of infertility cases are attributed to ovulatory disorders, with 70% of anovulatory women showing PCOS symptoms [5]. Studies indicate that spontaneous conception is delayed by an average of two years in PCOS patients, and the cumulative probability of delivery is significantly lower in these women than in non-PCOS individuals [6]. Furthermore, PCOS contributes to a significant increase in pregnancy-related complications, including early miscarriage, gestational diabetes mellitus, hypertensive disorders, and preeclampsia [7], and is significantly associated with an increased risk of endometrial cancer [8]. In summary, PCOS can exert substantial detrimental effects on both the physiological and psychological well-being of female patients.

At present, there remains no definitive cure for this disorder. Conventional medical management primarily focuses on symptom-specific interventions. For patients without fertility intentions, combined oral contraceptives (COCs) are administered to regulate menstrual cycles. Hyperandrogenism-related manifestations (*e.g.*, androgenic alopecia and hirsutism) are managed with anti-androgen agents such as cyproterone acetate/ethinylestradiol tablets to suppress androgen levels, or with spironolactone for androgen receptor blockade. In obese patients, insulin sensitizers like metformin are employed to reduce hyperinsulinemia and glycemic levels, while ovulation induction agents (*e.g.*, clomiphene citrate (CC) or letrozole) and surgical interventions are utilized for those pursuing pregnancy [9]. However, these pharmacotherapeutic strategies target isolated symptoms rather than the underlying pathogenesis, and treatment responses exhibit significant interindividual variability.

Traditional Chinese Medicine (TCM) has been extensively investigated as a potential complementary therapy for managing subfertility in women with PCOS. As an adjunctive treatment approach, traditional Chinese herbal therapy has shown considerable therapeutic potential, exhibiting favorable effects on glucose homeostasis, anti-inflammatory activity, and menstrual cycle regulation [10, 11]. A meta-analysis of 62 Bushen formulas revealed that Bushen-based therapies significantly improved ovarian mass, sex hormone profiles, and insulin resistance in rat models of PCOS [12]. Additionally, a meta-analysis of randomized controlled trials (RCTs) involving 609 women with PCOS-related infertility demonstrated that the combined use of TCM and CC may enhance pregnancy rates [13].

The Bushen Huatan Formula (BHF) is a Chinese herbal preparation developed based on the therapeutic principles of tonifying the kidneys, strengthening the spleen, eliminating dampness, and resolving phlegm. In dehydroepiandrosterone (DHEA)-induced PCOS rat models, BHF was shown to ameliorate disrupted estrous cycles, abnormal follicular development, elevated serum testosterone and insulin levels, as well as granulosa cell apoptosis [14]. Furthermore, in letrozole- and high-fat diet-induced PCOS mouse models, BHF improved intestinal barrier function by activating the PPARγ pathway in the gut, thereby contributing to PCOS management [15]. For obese PCOS patients, this comprehensive approach simultaneously addresses both the clinical manifestations and root pathogenesis of the disease.

Clinical studies have demonstrated that BHF can reduce inflammatory responses and oxidative stress in PCOS patients with hyperinsulinemia [16]. After a three-month intervention with BHF, significant reductions were observed in fasting insulin (FINS), homeostatic model assessment of insulin resistance (HOMA-IR), and triglyceride (TG) levels among patients. Furthermore, BHF was found to modulate clinical pregnancy outcomes in PCOS patients [17]. Treatment with this formula significantly improved the blastocyst formation rate and clinical pregnancy rate, while markedly reducing the incidence of early spontaneous abortion [18]. These findings collectively indicate that BHF modulates PCOS symptoms through multiple mechanisms.

Despite these promising results, there remains a lack of comprehensive synthesis regarding BHF’s efficacy in PCOS treatment. Therefore, this study conducted a meta-analysis of randomized controlled trials to evaluate the therapeutic potential of BHF and assess its role as a complementary therapy for PCOS.

## 2 MATERIALS AND METHODS

The present systematic review was conducted in accordance with the PRISMA 2020 guidelines, the Cochrane Handbook for Systematic Reviews of Interventions [19, 20], and the 2023 International Guideline on Polycystic Ovary Syndrome [21]. The protocol was registered on PROSPERO (ID: CRD42024564166).

### 2.1 Search Strategy

A comprehensive literature search was conducted across the following databases from their inception to June 20, 2024: Cochrane Library, Embase, PubMed, Web of Science, China National Knowledge Infrastructure (CNKI), Wanfang Database, and China Science and Technology Journal Database (VIP). The search strategy combined terms for the condition, intervention, and study type as follows:

PCOS-related terms: “polycystic ovary syndrome”, “PCOS”, “PCOD”, “PCO”, “Stein-Leventhal Syndrome”, “Stein Leventhal”, “ovary polycystic disease”, “Sclerocystic Ovary Syndrome”, “Sclerocystic Ovary Degeneration”, Ovary Degeneration”.

BHF-related terms: “Bushen Huatan formula”, “Bushen Huatan Prescription”, “Bushen Huatan recipe”, “Bushen Huatan”, “BushenHuatan”, “Bushen-huatan”.

RCT-related terms: “randomized controlled trial”, “randomized controlled trials”, “randomized controlled trial”, “RCT”, “randomization”, “randomly”, “clinical trial”.

Boolean operators were used to combine terms, and no language restrictions were applied.

### 2.2 Inclusion Criteria

Studies were eligible for inclusion in this systematic review based on the following PICOS framework:

Population (P): Patients diagnosed with PCOS according to established criteria, defined by the presence of at least two of the following: (i) clinical and/or biochemical hyperandrogenism (elevated total testosterone, free androgen index, or bioavailable testosterone); (ii) ovulatory dysfunction (oligo- or anovulation evidenced by irregular or absent menses); or (iii) polycystic ovarian morphology (multiple small antral follicles, ovarian enlargement, and/or elevated anti-Müllerian hormone levels).

Intervention (I): Bushen Huatan Formula (BHF) combined with conventional pharmacotherapy.

Comparison (C): Conventional pharmacotherapy alone.

Outcomes (O): Primary endpoints included ovulation rate and pregnancy rate; secondary endpoints included serum levels of testosterone (T), follicle-stimulating hormone (FSH), luteinizing hormone (LH), estradiol (E2), and endometrial thickness.

Study design (S): Randomized controlled trials (RCTs).

To ensure accuracy in data extraction and minimize interpretation bias, only studies published in English or Chinese were considered.

### 2.3 Exclusion Criteria

Studies were excluded if they met any of the following criteria: (1) Studies with titles, abstracts, or full texts irrelevant to the topic of this review; (2) Literature types such as reviews, conference papers, or animal studies; (3) Studies lacking key outcome measures.

### 2.4 Literature Selection and Data Extraction

Two researchers (Yaru Wang and Qingrui Zhang) independently performed the literature screening and data extraction using a predefined data collection form. The process was conducted in EndNote 20 and Microsoft Excel to manage references and extracted data. Any discrepancies between the two reviewers regarding study inclusion or data interpretation were resolved through discussion until a consensus was reached. In cases where agreement could not be achieved, a third senior reviewer (Shuai Zhang) was consulted to make the final decision. The following data were extracted from each included study: first author, year of publication, diagnostic criteria used, sample sizes in the trial and control groups, mean age of participants, intervention protocols for both groups, treatment duration, follow-up period, outcome measures (both primary and secondary), and records of adverse events.

### 2.5 Risk of Bias Assessment

Two researchers assessed the risk of bias in included studies using the Cochrane Collaboration's Risk of Bias Tool [22]. The studies were evaluated according to the following criteria: randomized sequence generation, allocation sequence concealment, blinding of subjects and staff, blinding of outcome assessment, incomplete outcome data, selective reporting of outcomes, and other sources of bias and classified as having a low, high, or unclear risk of bias. In the case of these studies, a third researcher undertook a review of the inconclusive studies.

### 2.6 Statistical Analysis

Statistical analyses were performed using RevMan 5.4. For dichotomous and continuous variables, odds ratios (ORs), standardized mean differences (SMDs), and 95% confidence intervals (CIs) were calculated. The heterogeneity of included studies was assessed using the I^2^ statistic and Chi-square test, with I^2^ values interpreted as follows: 0 to 100% (I^2^ = 0%–40%, probably low heterogeneity; I^2^ = 30%-60%, probably representing moderate heterogeneity; I^2^ = 50%–90%, probably representing high heterogeneity; and I^2^ = 75%–100%, considerable heterogeneity). If heterogeneity was significant (I^2^ > 50%, *P* < 0.05), a random-effects model was applied; otherwise, a fixed-effects model was used. Subgroup analyses were conducted to explore potential sources of heterogeneity, and sensitivity analyses were performed by omitting one study at a time to test result stability. Publication bias was evaluated using funnel plots and Egger’s test for datasets with ≥10 RCTs. Graphing was performed using R software. Evidence quality was assessed using the GRADE system, which evaluates bias, imprecision, inconsistency, indirectness, and publication bias. Evidence was rated as very low, low, moderate, or high certainty [23].

## 3 RESULTS

### 3.1 Study Selection

The initial search identified 371 articles, of which 164 were duplicates and subsequently excluded. After screening the remaining 207 articles for relevance, 70 articles were retained for further review. Full-text screening of these 70 articles was conducted to assess eligibility, resulting in the inclusion of 11 studies in this meta-analysis. The specific screening process is illustrated in Fig. (**1**).

### 3.2 Study Characteristics

A total of 11 randomized controlled trials involving 1,006 patients diagnosed with PCOS were included in the analysis. In the control group, biomedicines were categorized into two types: ovulation promoters, including metformin, letrozole, clomiphene citrate (CC), and human chorionic gonadotropin (HCG); and menstrual cycle modifiers, such as deoxypregnenate ethinyl E2, deoxypregnenate acetate tablets, compounded cyproterone acetate(CCA), and ethinyl E2 acetate cyproterone tablets. The intervention group received the same treatments as the control group, with the addition of BHF. Table **1** presents detailed information on the age and sample sizes of the two groups. Table **2** provides the Latin names and dosages of the Chinese medicines used for tonifying the kidney and resolving phlegm in the 11 studies. Table **S1** lists the frequency of use, medicinal parts, and sources of each Chinese medicine.

### 3.3 Quality Assessment

The 11 included studies demonstrated an overall low to moderate risk of bias. Regarding random sequence generation, eight studies employed the random number table method, one study used a lottery system to minimize selection bias [24], and two studies [25, 26] only described the process as random without specifying the method used, which resulted in their classification as unclear. In terms of allocation sequence concealment, none of the 11 studies reported whether concealment was implemented, leading to all being categorized as unclear. None of the studies mentioned blinding of investigators or participants, which resulted in all being judged as having a high risk of performance bias. However, for outcome assessment, all studies were considered to have a low risk of detection bias, as the measured outcomes were objective indicators, and the absence of blinding was unlikely to have influenced the results. Selective reporting bias was categorized as unclear for all studies due to the lack of access to their original protocols. Finally, all 11 studies were assessed as having a low risk of other biases.

### 3.4 Primary and Secondary Outcomes

#### 3.4.1 Pregnancy Rate

**Fig. (2):**
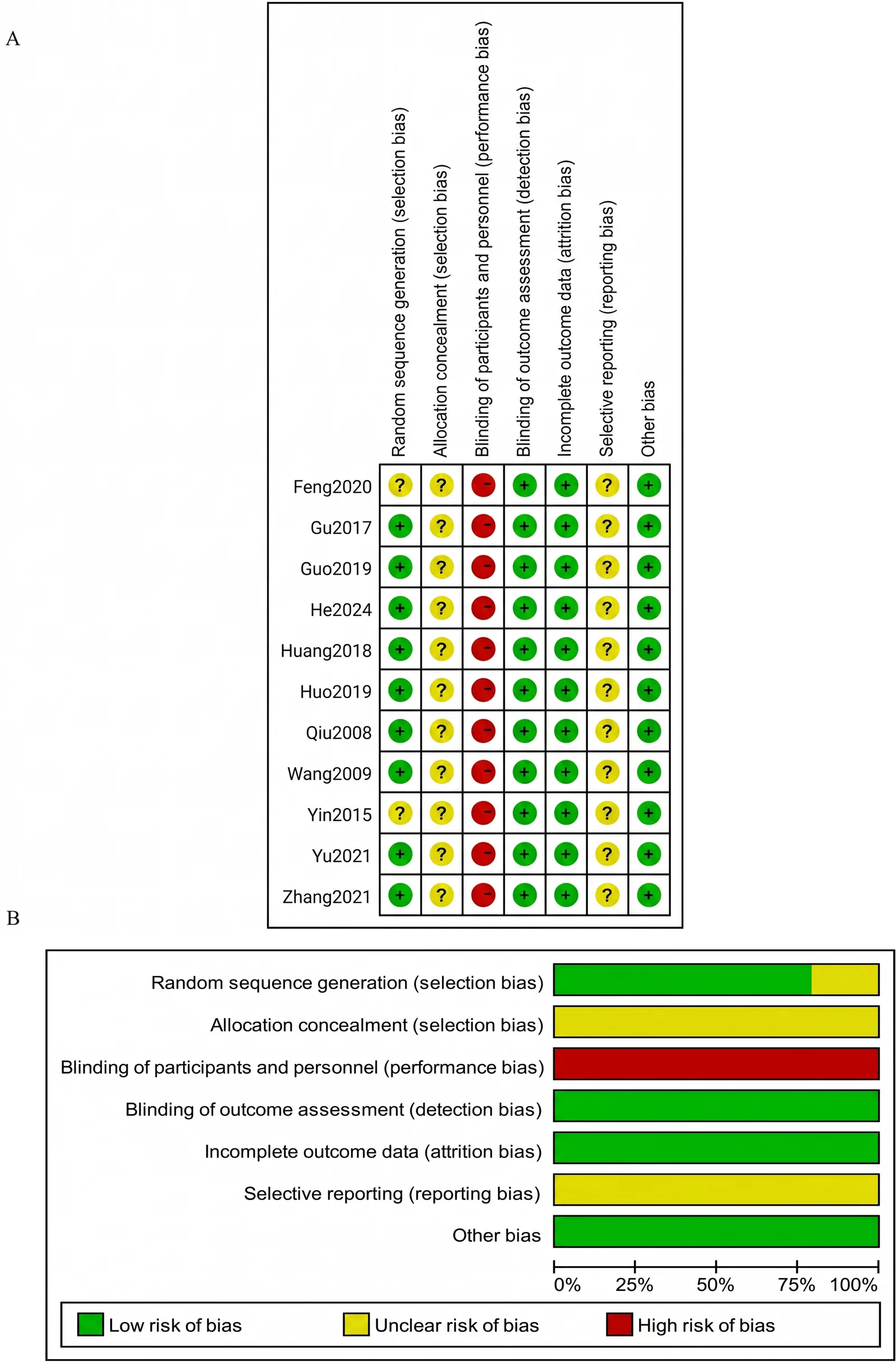
Forest plot of pregnancy rate between intervention and control groups.

All 11 studies reported pregnancy outcomes for both groups. The intervention group demonstrated a higher pregnancy rate compared to the control group, with the difference being statistically significant in nine studies but not in two [26, 27]. No heterogeneity was detected among the studies (*P* = 0.86, I^2^ = 0%), leading to the use of a fixed-effects model for pooled analysis. The pooled analysis demonstrated a significantly higher pregnancy rate in the BHF combination group (OR = 2.58, 95% CI: 1.97–3.38, *P* < 0.01; Fig. **2**) with no heterogeneity (I^2^ = 0%).

#### 3.4.2 Ovulation Rate

**Fig. (3):**
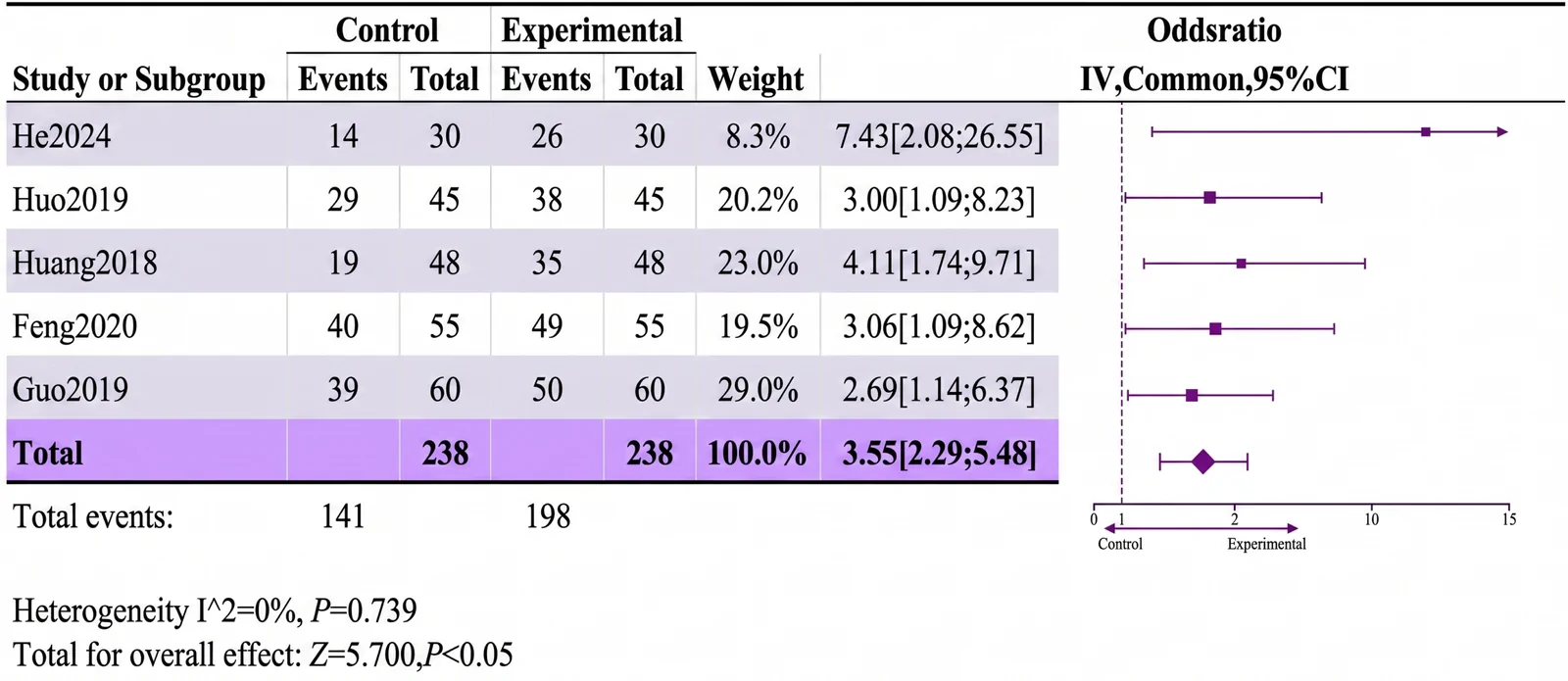
Forest plot of ovulation rate between intervention and control groups.

Five studies [24, 25, 27-29] reported ovulation outcomes. The intervention group had a significantly higher ovulation rate than the control group. As no heterogeneity was observed (*P* = 0.74, I^2^ = 0%), a fixed-effects model was applied. The pooled results showed a statistically significant difference in ovulation rates (OR = 3.55, 95% CI = 2.29 to 5.48, *P* < 0.01) (Fig. **3**). This indicates that combining BHF with conventional therapy is more effective than using conventional therapy alone for enhancing ovulation rates.

#### 3.4.3 Testosterone

**Fig. (4):**
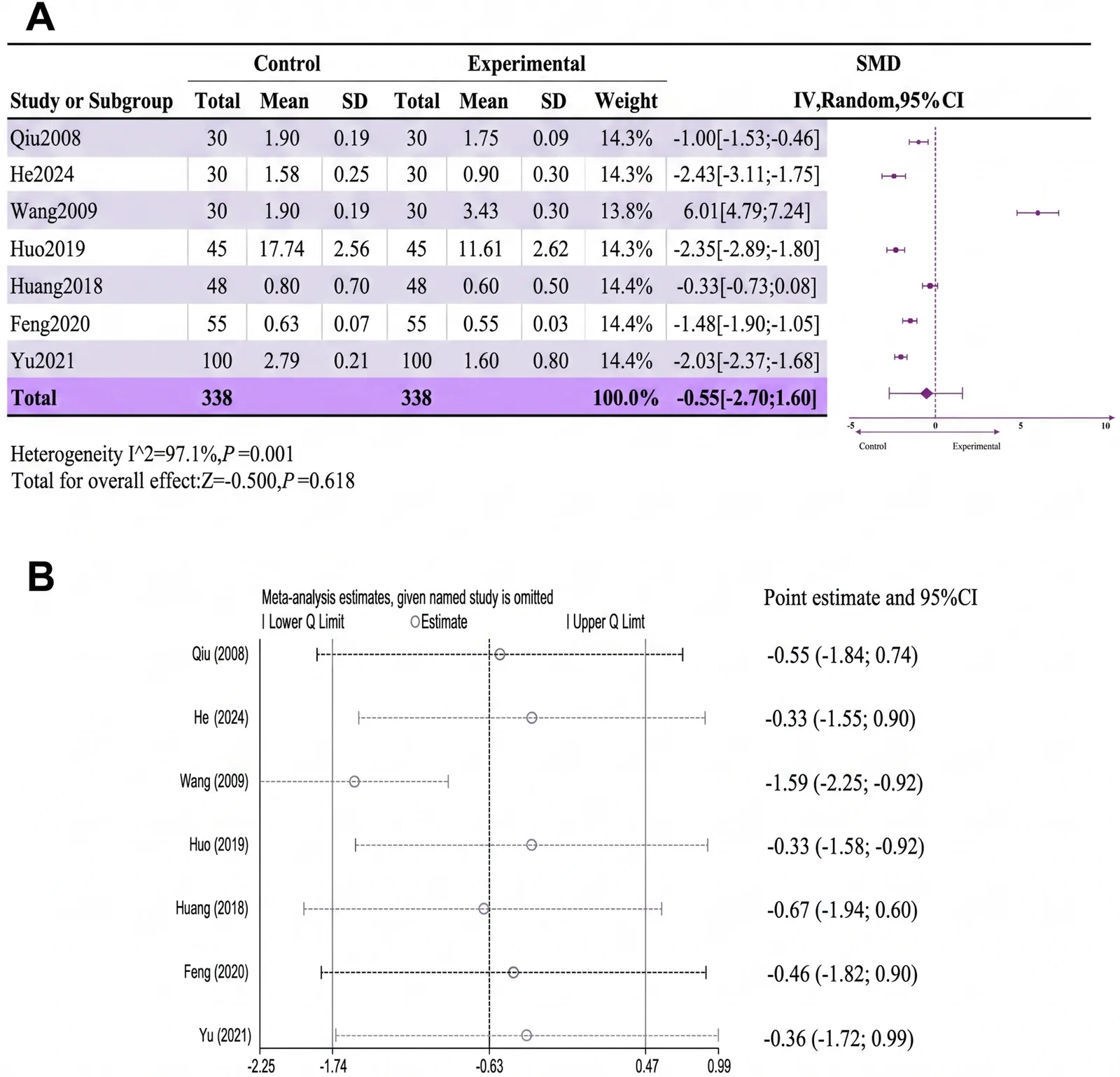
Meta-analysis of changes in testosterone levels in PCOS patients treated with BHF in combination with conventional drugs. (**A**) Forest plot of changes in testosterone levels after BMF in combination with conventional drugs. (**B**) Sensitivity analysis demonstrating the stability of the original testosterone results.

Seven randomized controlled trials [24, 25, 27, 28, 30-32] involving 676 participants per group evaluated T levels (Fig. **4A**). The meta-analysis revealed no significant between-group difference (SMD=-0.55, 95%CI:-2.70 to 1.60, *P*=0.62) using a random-effects model (I^2^=97.1%, *P*<0.01), indicating BHF combination therapy did not significantly alter T levels compared to conventional therapy alone. Three prespecified subgroup analyses were conducted based on control intervention types: (1)BHF + ovulation inducer *vs*. ovulation inducer alone (n=3 studies); BHF + menstrual cycle regulator *vs*. regulator alone (n=2 studies); BHF + combined (inducer + regulator) *vs*. combined therapy alone (n=2 studies). Subgroup differences were nonsignificant, with persistent heterogeneity (I^2^>50% in all subgroups; Fig. **S1A**). Sensitivity analysis using the leave-one-out method demonstrated that no single study disproportionately influenced the overall effect size (Fig. **4B**), suggesting the results were robust despite unexplained heterogeneity. The substantial unexplained heterogeneity may reflect variations in: (1) BHF composition across trials, (2) treatment duration, or (3) baseline T levels of participants.

#### 3.4.4 LH

**Fig. (5):**
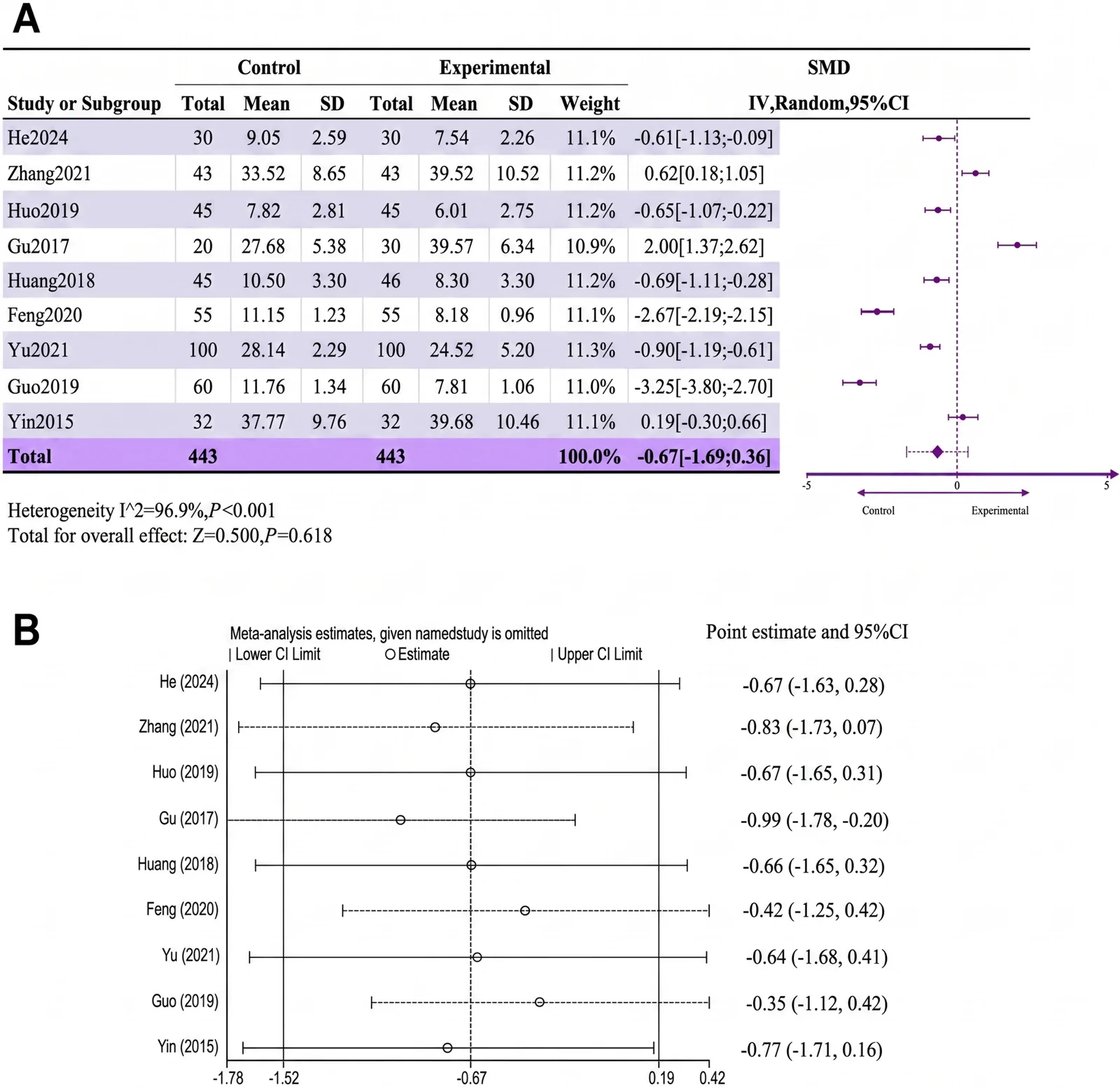
Meta-analysis of changes in LH levels in PCOS patients treated with BHF in combination with conventional drugs. (**A**) Forest plot of changes in LH levels after BMF in combination with conventional drugs. (**B**) Sensitivity analysis demonstrating the stability of the raw LH results.

Nine randomized controlled trials [24-29, 32-34] involving 886 participants compared changes in LH levels between the BHF combination therapy and conventional treatment alone. The pooled analysis using a random-effects model (due to significant heterogeneity: I^2^=96.9%, *P*<0.01) showed no statistically significant difference between groups (SMD=-0.67, 95%CI:-1.69 to 0.36, *P*=0.618; Fig. **5A**). Subgroup analyses by control intervention type (*e.g.*, ovulation induction *vs*. cycle regulation; Fig. **S1B**) and sensitivity analyses (Fig. **5B**) confirmed the robustness of this null association, though heterogeneity remained unresolved (see the above contents for potential sources).

#### 3.4.5 FSH

**Fig. (6):**
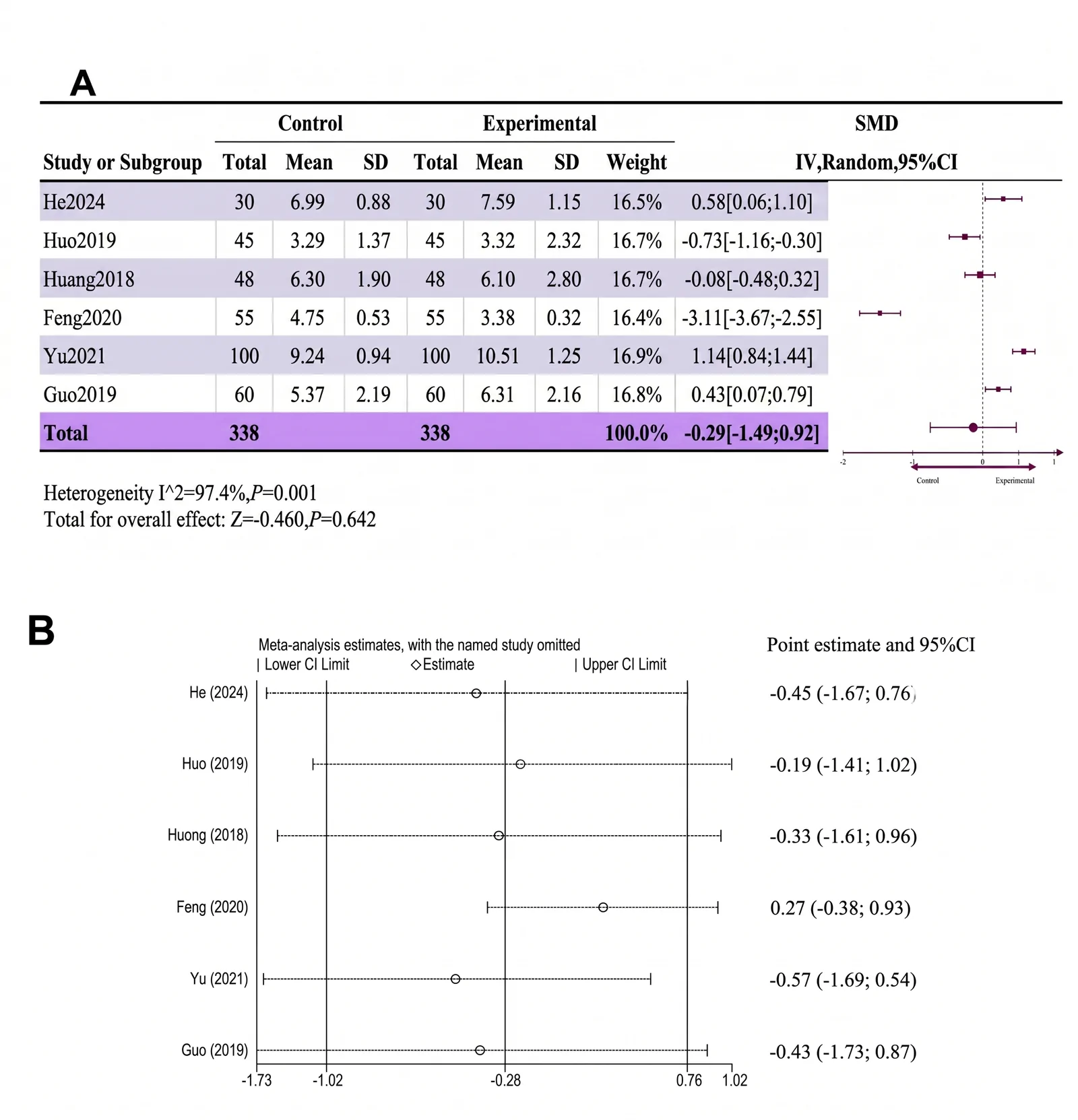
Meta-analysis of changes in FSH levels in PCOS patients treated with BHF in combination with conventional drugs. (**A**) Forest plot of changes in FSH levels after BMF in combination with conventional drugs. (**B**) Sensitivity analysis demonstrating the stability of the raw FSH results.

Six studies [24, 25, 27-29, 32] evaluating FSH levels showed no significant difference between BHF combination therapy and conventional treatment (SMD=-0.29, 95%CI:-1.49 to 0.92, *P*=0.64; random-effects model, I^2^=97.4%). Subgroup and sensitivity analyses confirmed result stability despite persistent heterogeneity (Figs. **6A**, **6B** and **S1C**).

#### 3.4.6 E2

**Fig. (7):**
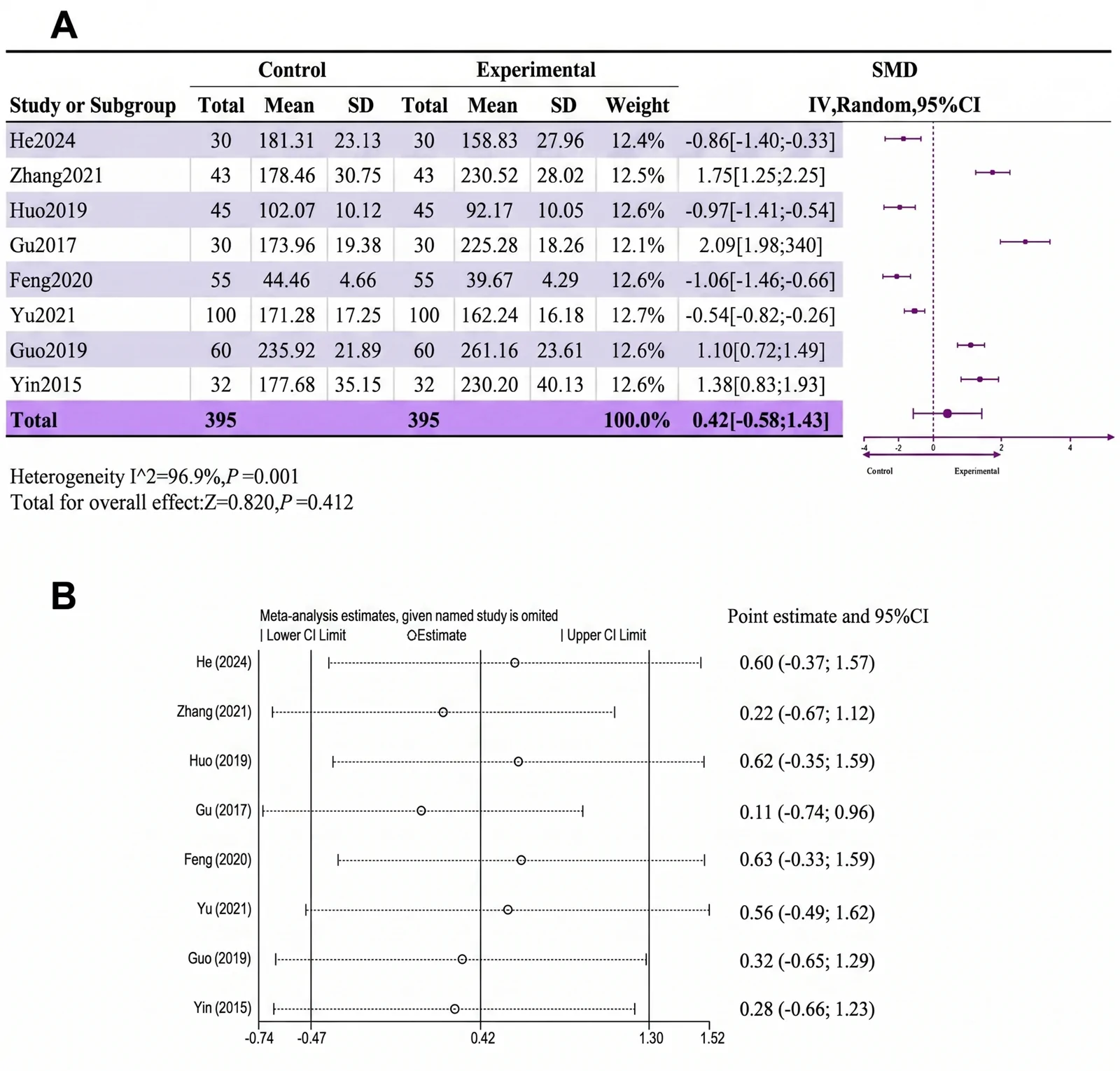
Meta-analysis of changes in E2 levels in PCOS patients treated with BHF in combination with conventional drugs. (**A**) Forest plot of changes in E2 levels after BMF in combination with conventional drugs. (**B**) Sensitivity analysis demonstrating the stability of the raw E2 results.

Eight RCTs [25-29, 32-34] assessing E2 levels showed no significant treatment effect (SMD=0.42, 95%CI:-0.58 to 1.43, *P*=0.41) using a random-effects model (I^2^=96.9%). Subgroup and sensitivity analyses confirmed the robustness of this finding despite substantial unexplained heterogeneity (Figs. **7A**, **7B** and **S1D**).

#### 3.4.7 Endometrial Thickness

**Fig. (8):**
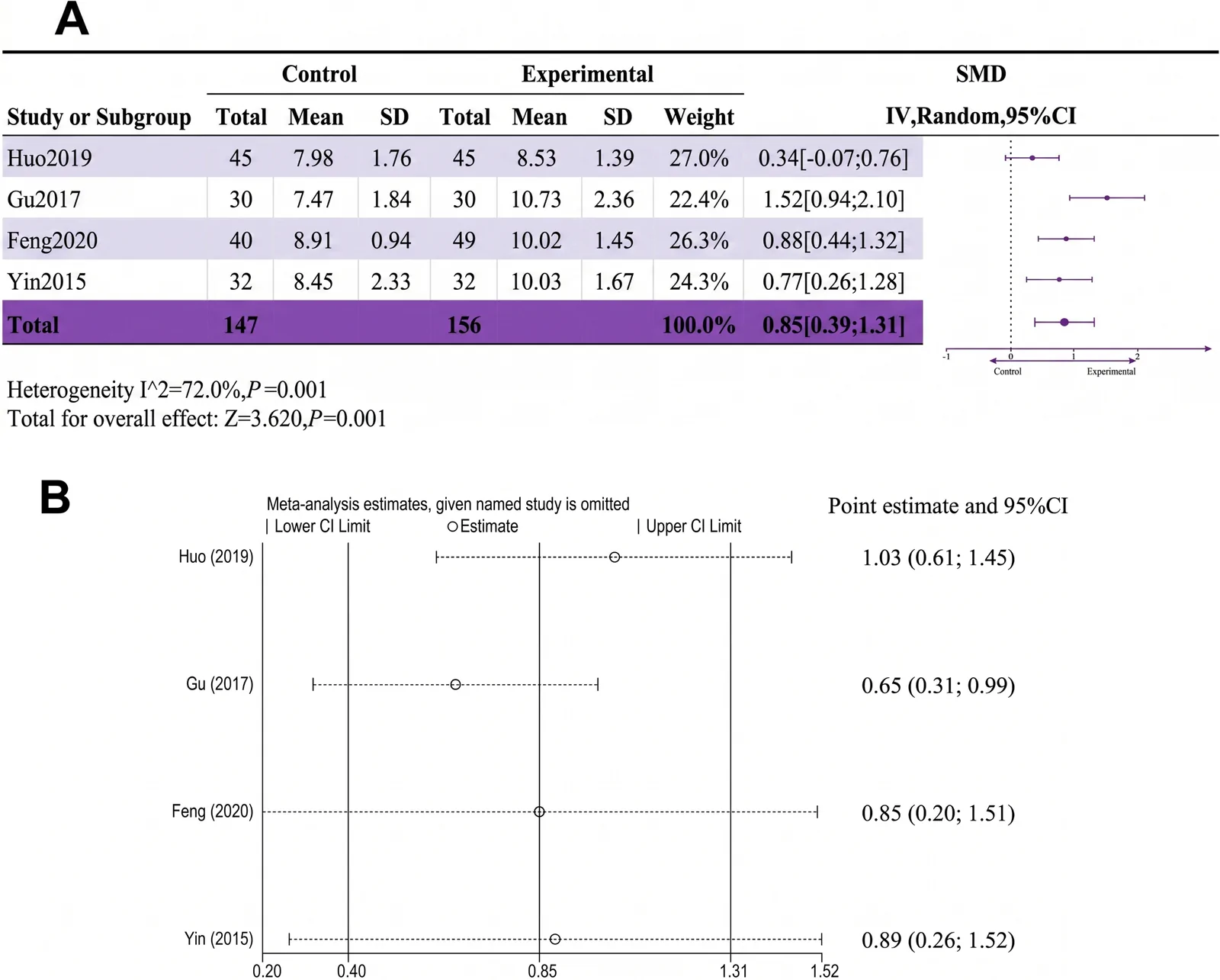
Meta-analysis of changes in EMTs levels in PCOS patients treated with BHF in combination with conventional drugs. (**A**) Forest plot of changes in EMTs levels after BMF in combination with conventional drugs. (**B**) Sensitivity analysis demonstrating the stability of the raw EMTs results.

Pooled analysis of four RCTs [25-27, 34] demonstrated significantly greater EMT improvement with BHF combination therapy *versus* conventional treatment alone (SMD=0.85, 95%CI:0.39-1.31, *P*=0.001; random-effects model, I^2^=72%). This treatment benefit remained robust in sensitivity analyses (Figs. **8A** and **8B**), though subgroup analyses by control intervention type failed to explain the observed heterogeneity (Fig. **S1E**).

#### 3.4.8 Adverse Effects

Safety data were reported in 2 of 11 included trials. One trial observed no adverse events in either group, while the other reported a lower incidence in the BHF combination group (3.33% *vs* 5.0% in controls). No significant between- group differences or serious adverse events were documented.

### 3.5 Publication Bias

**Fig. (9):**
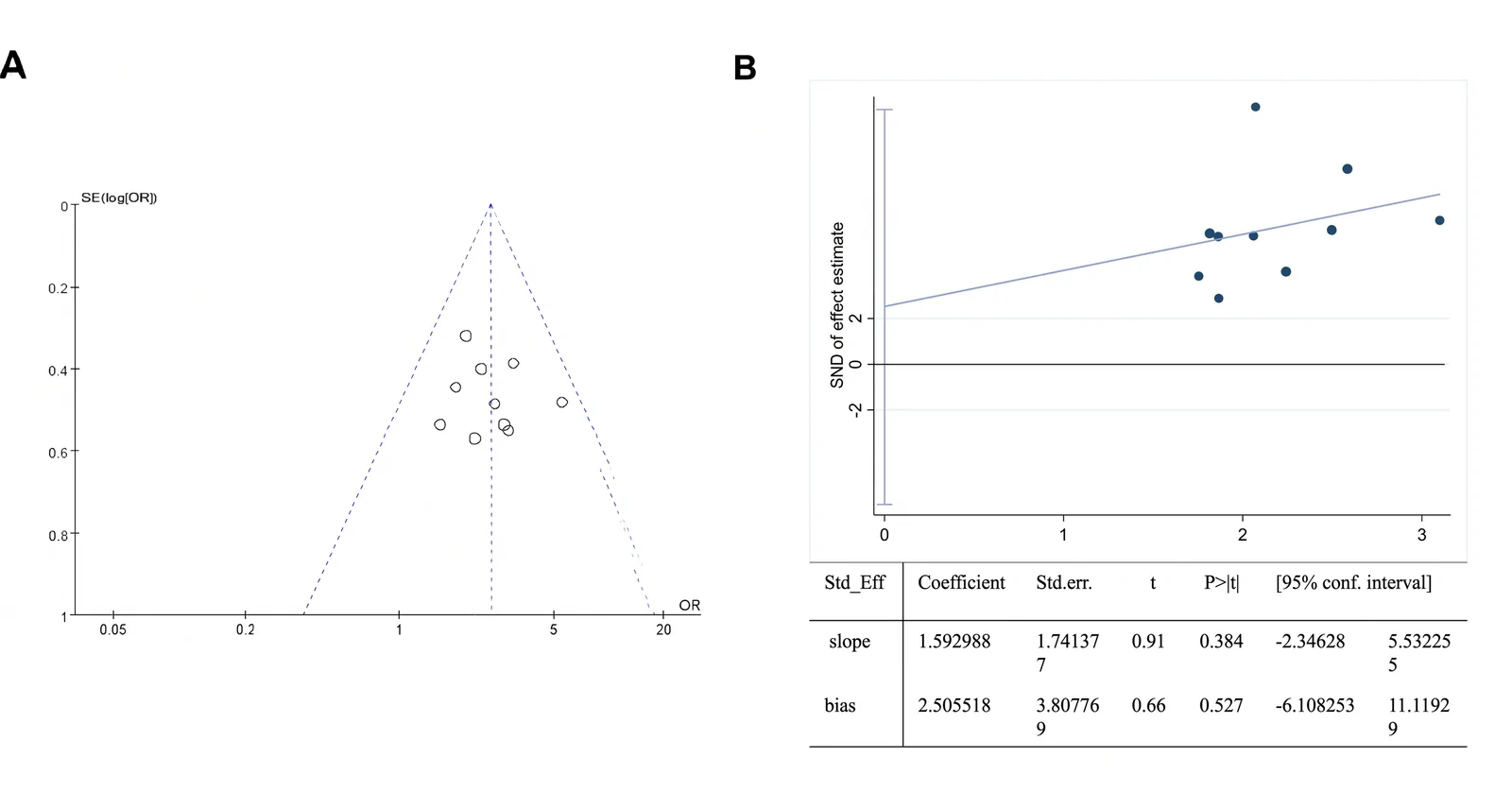
(**A**) Publication of offset funnel plots of pregnancy rates. (**B**) Egger's funnel plot of pregnancy rates.

Publication bias was assessed for pregnancy rates (the only outcome with ≥10 trials). Both funnel plot symmetry (Fig. **9A**) and Egger's test (*P*=0.527; Fig. **9B**) indicated low risk of publication bias.

### 3.6 Quality of Evidence

GRADE assessment yielded moderate-quality evidence for pregnancy and ovulation rates (downgraded one level for risk of bias from unblinded designs). For hormonal outcomes (T, LH, FSH, E2) and EMT, evidence was rated very low due to: (1) risk of bias (unblinding; -1 level), (2) substantial heterogeneity (I^2^>90% for T/LH/FSH, -2 levels; I^2^>50% for EMT, -1 level), and (3) imprecision (CI crossing equipoise: -1 level for T/LH/FSH; additional -1 level for EMT with n<400). See Table **3** for detailed ratings.

High certainty: we are very confident that the true effect lies close to that of the estimate of the effect.

Moderate certainty: we are moderately confident in the effect estimate: the true effect is likely to be close to the estimate of the effect, but there is a possibility that it is substantially different.

Low certainty: our confidence in the effect estimate is limited: the true effect may be substantially different from the estimate of the effect.

Very low certainty: we have very little confidence in the effect estimate: the true effect is likely to be substantially different from the estimate of effect.

## 4 DISCUSSION

### 4.1 Outcome Index Analysis

This systematic review and meta-analysis suggests that the addition of BHF to conventional treatment may be associated with improvements in clinical pregnancy rates (OR=2.58) and ovulation rates (OR=3.55), outcomes which showed no heterogeneity (I^2^=0%) but are supported by evidence of moderate certainty per GRADE assessment. The therapy may also enhance endometrial thickness (SMD=0.85), a potential mechanism for its effect on fertility outcomes. However, it is critical to interpret these findings in the context of the included studies' methodological limitations, which include a substantial risk of bias due to lack of blinding and unclear allocation concealment. No statistically significant changes were observed in hormonal levels (T, LH, FSH, E2), a finding accompanied by substantial between-study heterogeneity (I^2^>90%) and a GRADE assessment of very low-quality evidence for these outcomes. Regarding safety, the evidence is insufficient to draw definitive conclusions due to the limited reporting of adverse events; only 2 of the 11 included studies provided safety data. Within these two studies, the observed incidence of adverse events was lower in the BHF group (3.33%) compared to the conventional treatment group (5.0%), and no serious adverse events were reported. However, these limited findings require confirmation by future studies with more rigorous and comprehensive safety monitoring. Therefore, based on the current limited certainty of evidence, BHF may be considered a potential complementary therapy rather than a standalone treatment for PCOS management. The promising findings related to fertility parameters require confirmation by future large-scale, rigorously designed RCTs with robust blinding procedures, comprehensive safety monitoring, and detailed reporting to allow for a more definitive assessment of its efficacy, safety, and mechanisms of action.

From a modern pharmacological perspective, BHF likely exerts its therapeutic effects through multi-target mechanisms. The kidney-tonifying components (*e.g.*, *Cuscuta chinensis* Lam.) may modulate the hypothalamic-pituitary-ovarian (HPO) axis to improve follicular development [35]. Contemporary pharmacological investigations have demonstrated that the flavonoid constituents present in Cuscuta chinensis may facilitate dominant follicle selection through modulation of FSH receptor expression [36, 37]. Lycium barbarum polysaccharides can ameliorate oocyte quality by reducing 8-hydroxy-2'-deoxyguanosine (8-OHdG) and lipid peroxidation levels in ovarian tissue, thereby decreasing the proportion of atretic follicles and significantly improving blastocyst formation rates [38]. Moreover, icariin may ameliorate hyperandrogenism by upregulating the expression of aromatase (CYP19), a key enzyme responsible for the conversion of androgens (*e.g.*, testosterone) to estrogens (*e.g.*, estradiol) [39]. In contrast, the phlegm-resolving herbal components (*e.g.*, *Pinellia ternata* and *Citrus reticulata pericarpium*) may optimize ovarian function by ameliorating insulin resistance and oxidative stress. Modern pharmacological investigations have revealed that bioactive compounds derived from *Citrus reticulata pericarpium* can improve lipid metabolism through the AMP-activated protein kinase (AMPK) signaling pathway [40]. As a key bioactive constituent of *Citrus reticulata*, nobiletin suppresses ovarian oxidative stress *via* the AMPK/SIRT1 signaling pathwayswhile promoting oocyte maturation and subsequent embryo developmental competence [41, 42]. Naringenin upregulates phosphorylated AKT (p-AKT) expression in ovarian tissue, resulting in improved ovulation rates while suppressing androgen production and follicular cyst formation [43]. The synergistic actions of BHF appear to operate through concurrent amelioration of endocrine dysfunction and modulation of the local ovarian microenvironment. This dual regulatory capacity may account for the observed enhancement in ovulation and pregnancy rates without substantial alterations in systemic hormone profiles. Of particular clinical relevance is BHF's demonstrated improvement in endometrial thickness - a distinguishing therapeutic advantage over conventional ovulation-inducing agents such as clomiphene citrate, which are known to adversely affect endometrial receptivity [44].

### 4.2 Methodological Limitations

Although this study provides supportive evidence for the potential of Bushen Huatan Formula (BHF) in treating polycystic ovary syndrome (PCOS), several methodological limitations warrant discussion. In the meta-analysis of key hormonal outcomes (T, LH, FSH, E2), we observed exceptionally high heterogeneity (I^2^ > 90%). Such a high degree of heterogeneity strongly suggests that the real differences between the included studies far exceed chance variation, which substantially undermines the reliability of the pooled effect estimates. Through in-depth analysis, we attribute this heterogeneity to multiple sources. First, clinical heterogeneity exists due to variations in diagnostic criteria and baseline patient characteristics (*e.g.*, age, BMI). Second, intervention heterogeneity arises because although the BHF formulations shared the same fundamental therapeutic principle (“tonifying kidney and resolving phlegm”), the specific herb composition, dosages, and sources of materials likely differed across studies due to variations in practitioner expertise. Finally, although subgroup analysis ruled out control interventions as a major source, other methodological variations- such as treatment duration and assay methods for hormone measurement—may also have contributed to the heterogeneity. It is noteworthy that although the pooled effect estimates suggested a trend, the high heterogeneity rendered all these findings statistically non-significant (*P* > 0.05). This result more likely reflects inconsistency in the direction of effects among original studies—possibly even mutual cancellation—rather than indicating absolute ineffectiveness of the intervention. Furthermore, this study only assessed short- term reproductive outcomes; important clinical endpoints such as live birth rates and long-term metabolic risk reduction were not evaluated.

### 4.3 Future Research Directions

Based on an analysis of the included studies, the most frequently used kidney-tonifying herbs included Epimedium, Cuscuta, and Morinda officinalis, while the core phlegm-resolving herbs consisted of Poria and Atractylodes. The considerable heterogeneity in the specific composition and dosage of BHF across current studies indicates that the pooled results of this review reflect the overall treatment principle of “tonifying the kidney and resolving phlegm” rather than the effect of a standardized formula. Therefore, a primary objective for future research should be the development and validation of a standardized core BHF formulation based on these high-frequency herbs to ensure consistency and reproducibility in therapeutic applications. Subsequently, well-designed high-quality RCTs with extended follow-up periods (≥12 months) should be implemented to systematically evaluate long-term clinical outcomes, such as live birth rates and incidence of metabolic syndrome. These trials should incorporate robust blinding procedures and comprehensive safety monitoring. Such methodological enhancements will effectively address current limitations related to heterogeneity in study design and variability in outcome measures, thereby generating more robust and high-quality evidence regarding the efficacy and safety of BHF for the management of PCOS.

## CONCLUSION

This meta-analysis demonstrates that BHF may serve as a complementary therapy for PCOS, potentially improving fertility outcomes such as ovulation and clinical pregnancy rates. However, significant heterogeneity in herbal composition, methodological limitations, and insufficient safety reporting preclude definitive conclusions. Future rigorously designed trials using standardized BHF formulations and assessing long-term outcomes are essential to establish its efficacy and safety.

## Figures and Tables

**Fig. (1):**
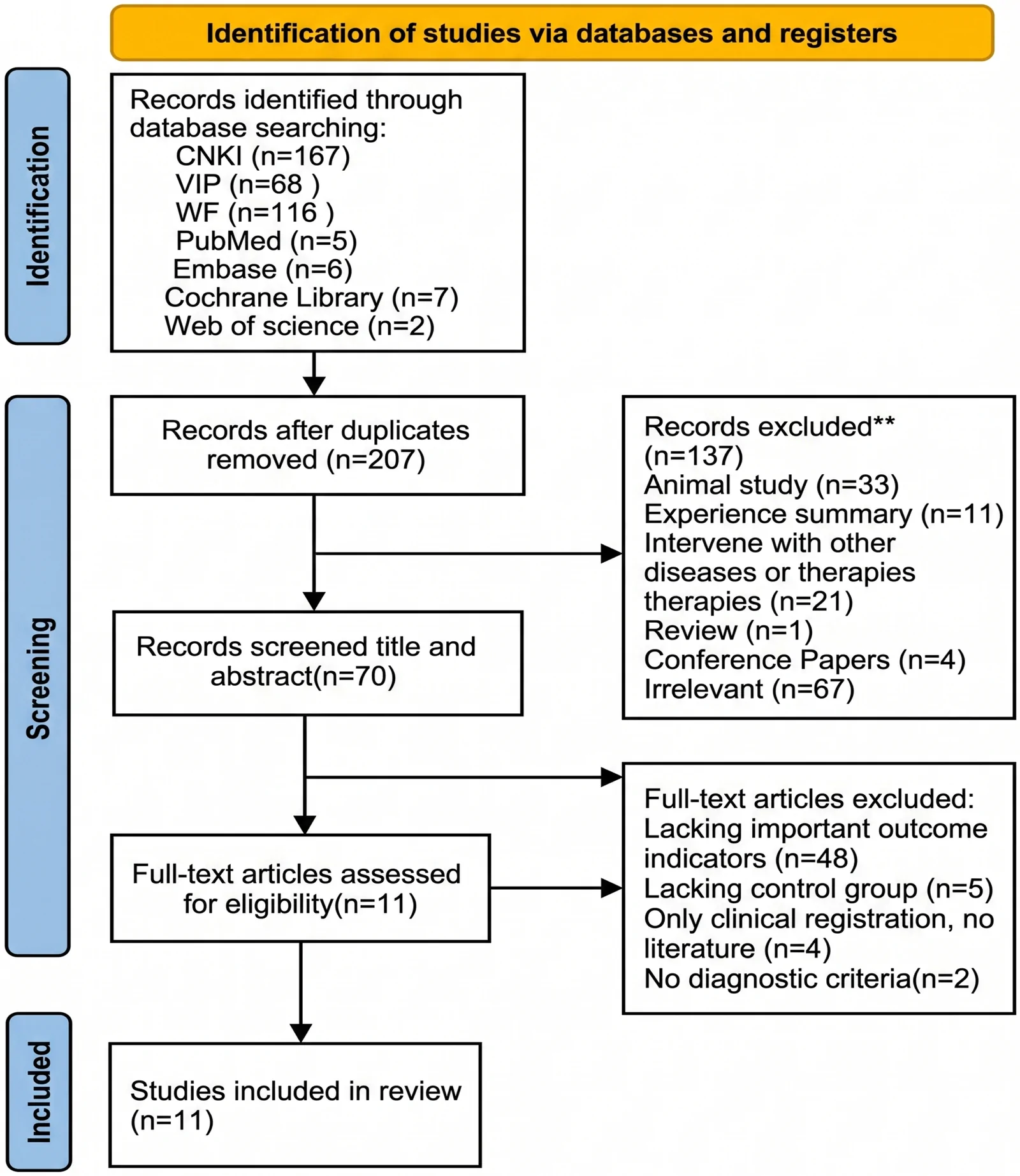
PRISMA flowchart.

**Table 1:** Specific characteristics of included studies.

Study ID	Diagnostic Criteria	Sample Size (I/C)	Age (years) (Mean±SD)	Intervention Group	Control Group	Duration/Follow-up	Outcome Measures	Adverse Event
Qiu2008	ESHRE/ASRM	30/30	I(27.03±3.81) C(26.67±3.81)	BHF, TCG	CCA/CC	3 months/6 months	1.Pregnancy rate 2.Hormonal levels (T)	NM
He2024	Chinese Consensus 2018	30/30	I(30.70±4.12) C(32.23±4.42)	BHF, TCG	MH	3 months/6 months	1.Ovulation and pregnancy rates 2. Hormonal levels (T, LH, FSH, E_2_)	NM
Wang2009	ESHRE/ASRM	30/30	I(27.03±3.81) C(26.67±3.81)	BHF, TCG	ECAT, CC	3 months/6 months	1.Ovulation and pregnancy rates 2. Hormonal levels (T)	NM
Zhang2021	Endocrine expert consensus	43/43	I(28.6± 3.4) C(28.5±3.5)	BHF, TCG	CA, Eth, let	3 months/ one year	1.Ovulation and pregnancy rates 2. Hormonal levels (LH, E_2_) 3. endometrial receptivity	NM
Huo2019	ESHRE/ASRM	45/45	I(28. 12 ± 3. 76) C(27. 67 ± 3. 57)	BHF, TCG	Let, HCG	5 day/NM	1.Ovulation and pregnancy rates 2. Hormonal levels (T, LH, FSH, E2) 3. endometrial receptivity	NM
Gu2017	Obstetrics and gynecology	30/30	I(30.5±2.3) C(33.5±1.9)	BHF, TCG	ECAT, CC	3 months/NM	1.Ovulation and pregnancy rates 2. Hormonal levels (LH, E2) 3. endometrial receptivity	NM
Huang2018	ESHRE/ASRM	48/48	I(58.2±6.6) C(57.3±8.3)	BHF, TCG	DE, Let, HCG	3 months/NM	1.Ovulation and pregnancy rates 2. Hormonal levels (T,LH, FSH) 3. endometrial receptivity	No
Feng2020	Obstetrics and gynecology	55/55	I(27.16±8.03) C(27.51±8.38)	BHF, TCG	CC	3 months/NM	1.Ovulation and pregnancy rates 2. Hormonal levels (T,LH, FSH, E_2_) 3. endometrial receptivity	NM
Yu2021	ESHRE/ASRM	100/100	I(33. 26±3.17) C(33.31±3.22)	BHF, TCG	CCA	3 months/one year	1pregnancy rate 2. Hormonal levels(T,LH, FSH, E2)	NM
Guo2019	ESHRE/ASRM	60/60	I(27.46±3.24) C(26.17±3.39)	BHF, TCG	met	3 months/one year	1.Ovulation and pregnancy rates 2. Hormonal levels (LH, FSH, E2)	I(2 cases) C(3 cases)
Yin2015	Obstetrics and gynecology	32/32	I(27.84±3.98) C(28.13±4.28)	BHF, TCG	Eth, Cyp, Let, HCG	I(108day) C(123day)/NM	1.pregnancy rate 2. Hormonal levels(LH, E2) 3. endometrial receptivity	NM

**Abbreviations:** CG: Treatment in control group; CCA: Compound cyproterone acetate; MH: metformin hydrochloride; ECAT: Ethinylestradiol and cyproterone acetate tablets; CA: Cyproterone acetate; Eth: Ethinylestradiol; Let: letrozole; DE: Desogestrel and Ethinylestradiol; Eth: Ethinylestradiol; Cyp: cyproterone; NM: Not mention.

**Table 2:** Composition of the BHF included in the study.

First Author	Year	Formula Name	TCM Composition
Qiu	2008	BHF	Morindae officinalis radix15g, Epimedii Polium15g, Cuscutae semen15g, Angelicae sinensis radix10g, Rehmanniae radix praeparata15g, Corni fructus15g, Cioscoreae rhizoma15g, Pinelliae rhizoma10g, Poria10g, Atractylodis rhizoma10g, Cyperi rhizoma6g, Salviae miltiorrhizae radix et rhizoma6g, Glycyrrhizae radix et rhizoma3g
He	2024	BHF	Pinelliae rhizoma10g, Citri reticulatae pericarpium10g, Poria10g, Codonopsis radix8g, Atractylodis macrocephalae rhizoma8g, Cuscutae semen15g, Morindae officinalis radix10g, Glycyrrhizae radix et rhizoma6g, Spatholobi caulis10g
Wang	2009	BHF	Morindae officinalis radix6g, Epimedii Polium12g, Dioscoreae rhizoma12g, Cuscutae semen12g, Rehmanniae radix praeparata12g, Corni fructus6g, Pinelliae rhizoma10g, Poria10g, Atractylodis rhizoma10g, Cyperi rhizoma6g, Salviae miltiorrhizae radix et rhizoma6g, Persicae semen12g, Glycyrrhizae radix et rhizoma3g
Zhang	2021	BHF	Astragali radix50g, Poria30, Salviae miltiorrhizae radix et rhizoma30g, Atractylodis rhizoma30g, Epimedii Polium30g
Huo	2019	BHF	Cyperi rhizoma18g, Pinelliae rhizoma18g, Cuscutae semen10g, Dipsaci radix10g, Cyathulae radix10g, Leonuri herba10g, Acori tatarinowii rhizome12g, Arisaematis rhizoma15g, Citri exocarpium rubrum18g, Poria15g, Citri reticulatae pericarpium10g, Angelicae sinensis radix12g, Salviae miltiorrhizae radix et rhizoma12g, Glycyrrhizae radix et rhizoma6g
Gu	2017	BHF	Astragali radix30g, Codonopsis radix20g, Poria20g, Atractylodis rhizoma20g, Salviae miltiorrhizae radix et rhizoma20g, Epimedii Polium20g, Morindae officinalis radix20g, Cyperi rhizoma20g, Cuscutae semen20g, Acori tatarinowii rhizome20g
Huang	2018	BHF	Rehmanniae radix praeparata24g, Dioscoreae rhizoma12g, Poria12g, Corni fructus9g, Alismatis rhizoma12g, Morindae officinalis radix12g, Curculiginis rhizoma9g, Polygonati rhizoma12g, Angelicae sinensis radix12g, Chuanxiong rhizoma12g, Pangolin9g, Citri reticulatae pericarpium9g, Arisaematis rhizoma12g, Gleditsiae spina12g, Glycyrrhizae radix et rhizoma6g
Feng	2020	BHF	Rehmanniae radix praeparata20g, Pinelliae rhizoma15g, Epimedii polium15g, Angelicae sinensis radix15g, Chuanxiong rhizoma15g, Cuscutae semen10g, Lycii fructus10g, Poria10g, Cyperi rhizoma10g, Dioscoreae rhizoma9g, Corni fructus9g, Acori tatarinowii rhizome9g, Salviae miltiorrhizae radix et rhizoma9g
Yu	2021	BHF	Epimedii Polium15g, Chuanxiong rhizoma15g, Corni fructus12g, Lycii fructus12g, Cyperi rhizoma12g, Cistanches herba9g, Angelicae sinensis radix12g, Salviae miltiorrhizae radix et rhizoma18g, Lycopi herba15g, Sparganii rhizoma8g, Curcumae rhizoma8g, Liquidambaris fructus12g, Citri reticulatae pericarpium9g, Glycyrrhizae radix et rhizoma6g
Guo	2019	BHF	Rehmanniae radix praeparata30g, Cistanches herba15g, Morindae officinalis radix15g, Epimedii Polium15g, Cuscutae semen15g, Dioscoreae rhizoma15g, Paeoniae radix rubra15g, Lycopi herba15g, Chuanxiong rhizoma15g, Leonuri herba15g, Corni fructus10g,Glycyrrhizae radix et rhizoma6g
Yin	2015	BHF	Salviae miltiorrhizae radix et rhizoma30g, Epimedii Polium30g, Astragali radix50g, Poria30g, Atractylodis rhizoma30g

**Table 3:** GRADE working group grades of evidence.

Outcomes	Effect		Certainty of the Evidence (GRADE)	
	Relative (95%CI)	Absolute		
Pregnancy rate	OR 2.58 (1.97 to3.38)	232 more per 1000 (from 166 more to 295 more)	1006(11 RCTs)	⊕⊕⊕○Moderate
		221 more per 1000 (from 154 more to 288 more)		
Ovulation rate	OR 3.55 (2.29 to 5.48)	245 more per 1000 (from 177 more to 296 more)	476(5 RCTs)	⊕⊕⊕○Moderate
		221 more per 1000 (from 162 more to 264 more)		
T	-	SMD 0.55 lower (2.70 lower to 1.60 higher)	676(7 RCTs)	⊕○○○ Very low
LH	-	SMD 0.67 lower (1.69 lower to 0.63 higher)	886(9 RCTs)	⊕○○○ Very low
FSH	-	SMD 0.29 lower (1.49 lower to 0.92 higher)	676(6 RCTs)	⊕○○○ Very low
E2	-	SMD 0.42 lower (0.58 lower to 1.43 higher)	790(8 RCTs)	⊕○○○ Very low
EMT	-	SMD 0.85 lower (0.39 lower to 1.31 higher)	303(4 RCTs)	⊕○○○ Very low

## Data Availability

All data generated or analyzed during this study are included in this published article.

## LIST OF ABBREVIATIONS

BHF: Bushen Huatan Formula
PCOS: Polycystic Ovary Syndrome
CNKI: China National Knowledge Infrastructure
RCTs: Randomized Controlled Trials
OR: Odds Ratios
SMDs: Standardized Mean Differences
CIs: Confidence Intervals
LH: Luteinizing Hormone
FSH: Follicle-Stimulating Hormone

## STANDARDS OF REPORTING

PRISMA guidelines and methodologies were followed.
